# Temporal variations in the gut microbial diversity in response to high-fat diet and exercise

**DOI:** 10.1038/s41598-024-52852-4

**Published:** 2024-02-08

**Authors:** Saba Imdad, Byunghun So, Junho Jang, Jinhan Park, Sam-Jun Lee, Jin-Hee Kim, Chounghun Kang

**Affiliations:** 1 Molecular Metabolism in Health and Disease, Exercise Physiology Laboratory, Sport Science Research Institute, Inha University, Incheon, 22212 South Korea; 2 Department of Biomedical Laboratory Science, College of Health Science, Cheongju University, Cheongju, 28503 South Korea; 3 Department of Sport Rehabilitation, College of Health, Welfare, and Education, Tong Myong University, Busan, 48520 South Korea; 4 Department of Physical Education, College of Education, Inha University, Incheon, 22212 South Korea

**Keywords:** Microbiology, Microbial communities

## Abstract

High-fat diet-induced obesity is a pandemic caused by an inactive lifestyle and increased consumption of Western diets and is a major risk factor for diabetes and cardiovascular diseases. In contrast, exercise can positively influence gut microbial diversity and is linked to a decreased inflammatory state. To understand the gut microbial variations associated with exercise and high-fat diet over time, we conducted a longitudinal study to examine the effect of covariates on gut microbial diversity and composition. Young mice were divided into four groups: Chow-diet (CHD), high-fat diet (HFD), high-fat diet + exercise (HFX), and exercise only (EXE) and underwent experimental intervention for 12 weeks. Fecal samples at week 0 and 12 were collected for DNA extraction, followed by 16S library preparation and sequencing. Data were analyzed using QIIME 2, R and MicrobiomeAnalyst. The Bacteroidetes-to-Firmicutes ratio decreased fivefold in the HFD and HFX groups compared to that in the CHD and EXE groups and increased in the EXE group over time. Alpha diversity was significantly increased in the EXE group longitudinally (*p* < 0.02), whereas diversity (Shannon, Faith’s PD, and Fisher) and richness (ACE) was significantly reduced in the HFD (*p* < 0.005) and HFX (*p* < 0.03) groups over time. Beta diversity, based on the Jaccard, Bray–Curtis, and unweighted UniFrac distance metrics, was significant among the groups. *Prevotella*, *Paraprevotella*, *Candidatus arthromitus*, *Lactobacillus salivarius*, *L. reuteri*, *Roseburia*, *Bacteroides uniformis*, *Sutterella*, and *Corynebacterium* were differentially abundant in the chow-diet groups (CHD and EXE). Exercise significantly reduced the proportion of taxa characteristic of a high-fat diet, including *Butyricimonas*, *Ruminococcus gnavus*, and *Mucispirillum schaedleri*. Diet, age, and exercise significantly contributed to explaining the bacterial community structure and diversity in the gut microbiota. Modulating the gut microbiota and maintaining its stability can lead to targeted microbiome therapies to manage chronic and recurrent diseases and infections.

## Introduction

Obesity is a complex multifactorial disease attributed to excessive adiposity, with 60% of adults and nearly one third of children affected by obesity and overweight issues. Overweight and obesity are the fourth most common risk factors for multiple noncommunicable diseases (NCDs), after elevated blood pressure, diet-related risks, and tobacco use^1^. Moreover, with reference to recent events, several meta-analyses and reports have documented an increased risk of COVID-19 morbidity and mortality linked to obesity and body mass index (BMI)^2–5^. NCDs, such as cardiovascular diseases, type 2 diabetes (T2D), hepatic steatosis, cancer^6^, and chronic respiratory diseases, are categorized as chronic diseases caused by a combination of genetic, environmental, and lifestyle factors^7^. The adverse effects of the therapies for the management of NCDs or chronic diseases associated with high-fat diet and obesity have refocused research into bacteriotherapy or microbiome therapy^8^.

Human beings consist of trillions of symbiotic microbes and bacteria, whose collective genomes make up the microbiome^9^. The microbiome has coevolved with the human host and shapes the physiological well-being of the host by actively impacting various host functions, such as energy metabolism, training and regulating the immune system, helping in digestion, producing vitamins and other essential compounds, including antimicrobials, and protecting against harmful pathogens^10^. The bulk bacterial metabolites and complex bacteria–host interactions form an active and diverse microbial ecosystem in the gut that plays a crucial role in maintaining health and preventing diseases^11^. The gut microbiota, with a repertoire of bacteria and other microbes, is dynamic and resilient to variations through influencing factors that are in constant flow. However, apart from natural fluctuations, genetics^12^, intestinal mucosa^13^, the immune system^14^, native microbiota, physical activity or exercise^15^, diet^16^, and exposure to antibiotics and other medications are the major factors influencing homeostasis and gut dysbiosis^17–19^. Deviations in the gut microbiota can lead to negative health impacts and an increased risk of NCDs in general as well as gastrointestinal diseases, including inflammatory bowel disease (IBD)^20^ and celiac disease^21^, in particular.

The gut microbiome has been extensively studied and has been associated with obesity and obesity-related disorders^22–27^. Previous reports have shown that a high-fat diet intake can impair the gut microbial community, leading to systemic inflammation and insulin resistance, thus linking obesity to insulin resistance^28–30^. In contrast, exercise is associated with the physiological well-being of humans by influencing microbial diversity, modulating mucosal immunity, and improving barrier function, potentially contributing to weight reduction, improved gut health, and a reduced risk of metabolic diseases^19,31–33^. Exercise is known to influence whole-body insulin sensitivity, which is mediated by enhanced insulin-dependent and independent glucose uptake by skeletal muscles^34^. Regular exercise has the potential to mitigate the detrimental effects of a high-fat diet and aid in weight management. A recent systematic review showed that one hour of aerobic training and physical activity (at 60% maximum heart rate [HRmax]) influenced beta diversity in athletes, whereas the abundance of beneficial microbial metabolites, short-chain fatty acids (SCFAs), was markedly influenced by increased physical activity in nonathletes, highlighting a more diverse intestinal microbiota in athletes^35^.

Multiple studies have elaborated on the effect of a range of factors, including high-fat diet and exercise, on the composition of the gut microbiome; however, most studies are cross-sectional in nature, and the comprehension of the intervention dynamics associated with the gut microbiota is limited. Here, we performed a longitudinal analysis to account for temporal changes in the composition, stability, and diversity of the gut microbiota over a period of 12 weeks, with high-fat diet (chow control diet) and treadmill exercise (sedentary control) interventions in a diet-induced mouse model of obesity.

## Materials and methods

### Animal care

Four-week-old C57BL/6 J female mice were housed in sterile cages at a temperature of 22 ± 2 °C and relative humidity of 50 ± 10%, under a 12 h light–dark cycle, with ad libitum access to food and water. Animal experiments were authorized by the Ethics Committee of Inha University (INHA 220,203–811, 2022–02-03) and were conducted in accordance with the relevant regulations. The reported methods and outcomes comply with the ARRIVE (Animal Research: Reporting of In Vivo Experiments) guidelines.

### Diet intervention and exercise training

For the 12-week longitudinal study, 20 mice were randomly assigned to four intervention groups. The sedentary groups were fed either control chow diet (CHD; fat 4%) (rodent NIH-31 open formula auto diet; Zeigler Feed) or high-fat diet (HFD; fat 60%) (D12492; Research Diets). The exercise groups performed treadmill running with intake of either chow diet (EXE) or high fat-diet (HFX) for 5 days a week. Prior to starting the exercise experiment, the mice were acclimatized to the treadmill exercise for one week. The EXE and HFX groups underwent daily exercise training for 1 h in a treadmill chamber, at a 5% incline and an intensity starting at 4 m/min. Exercise intensity was increased to 15 m/min for the first half of the training period and to 20 m/min for the second half, followed by a cool-down period (5 m/min for 5 min). The weight of the mice was recorded weekly until the final week. After completion of the experiment, the mice were euthanized by isoflurane overdose in random order.

### Sample collection and DNA extraction

Mice feces were collected by restraining the animals, whereby they were allowed to defecate individually in sterile containers. The fecal pellets were then transferred under sterile conditions in microcentrifuge tubes, snap frozen, and stored at − 80 °C, until further processing. Fecal samples were collected at two time points: at the beginning (week 0) and at the end of the experiment (week 12). The fecal samples were thawed at 4 °C and a sample of ~ 150 mg was homogenized in a Fast-Prep 24 (MP Biomedicals, Irvine, CA, USA) bead homogenizer for DNA extraction using the SPINeasy DNA Kit for Feces (MP Biomedicals), according to the manufacturer’s protocol. The extracted DNA was electrophoresed on a 1% agarose gel and visualized for quality using the ChemiDoc imaging system (Bio-Rad, Hercules, CA, USA). DNA quality was further examined using a SpectraMax iD3 spectrophotometer (Molecular Devices, San Jose, CA, USA) and quantified using Qubit 4 (Thermo Fisher Scientific, Waltham, MA, USA).

### 16S library preparation

A two-step PCR amplification protocol was used for DNA amplification. Briefly, in the first PCR run, the V4 region of the bacterial 16S rRNA gene was amplified using the 515F and 806R primers (F-primer: 5′-TCG TCG GCA GCG TCA GAT GTG TAT AAG AGA CAG GTG CCA GCM GCC GCG GTA A-3′ and R-primer: 5′- GTC TCG TGG GCT CGG AGA TGT GTA TAA GAG ACA G-3′) and the 2X KAPA Hifi HotStart ReadyMix (KAPA Biosystems, UK). The second PCR was run to attach dual indices and Illumina sequencing adapters, and libraries were generated using a Nextera XT Index Kit v2 (Illumina, San Diego, CA, USA). PCR products were cleaned using AMPure XP beads (Beckman Coulter, USA) after each PCR amplification. PCR thermal cycling conditions were as described in our previous publication^36^. Equal concentrations of purified amplicons were pooled, and paired-end sequencing was carried out on the iSeq 100 Illumina platform for 150 cycles at a targeted depth of 1.2 Gb.

### Data and statistical analyses

The paired-end raw reads were imported into QIIME 2 v2022.02^37^. Quality plots were produced using FastQC and inspected for quality control parameters, including trimming of reads, adapter sequences, and PhiX contamination. Cutadapt v4.0^38^ was used to remove the primers, and the reads were quality-filtered with a minimum median quality (Phred) score of 20. Since the joining of paired-end reads did not yield sufficient merged reads, the analysis was carried out with forward reads only. The reads were denoised using the Deblur plugin and trimmed at the 3′ end (19 bp) to remove low-quality bases, and the abundances and representative sequences were extracted. The phylogenetic tree was constructed using the fragment-insertion method, where the sequence variants were aligned using SEPP (SATé-enabled Phylogenetic Placement)^39^, against the Greengenes 13.8 reference database^40^. The same reference database was used to extract the 16S rRNA V4 region sequences to train the Naive Bayes classifier^41^ for taxonomic classification of representative sequences. Samples with fewer than 7570 sequences were removed during rarefaction for downstream analysis. Alpha diversity indices, including Shannon, Pielou’s evenness, and Faith’s phylogenetic diversity (PD) were estimated via QIIME 2. Moreover, the qiime2 output files were imported into R v4.2.2 using the qiime2R package v0.99.6 and microbial richness, including the abundance-based coverage estimator (ACE), Fisher, and Inverse Simpson indices, was estimated using the R phyloseq package v1.42.0. Šidák’s test and the improved Benjamini–Hochberg procedure for false discovery rate (FDR) correction were employed to calculate the significance of alpha diversity metrics across multiple groups. The beta diversity of the microbial communities was assessed using the Jaccard, Bray–Curtis, unweighted, and weighted UniFrac distance metrics, and principal coordinate analysis (PCoA) plots were generated to segregate the samples and determine community structure. The PCoA data were subjected to pairwise permutational multivariate analysis of variance (PERMANOVA) by comparing the true F statistics to the distribution of F statistics randomly permuted (default = 999) from the data. The PERMANOVA results were confirmed using PERMDISP. The significant covariates of microbial diversity were examined using ADONIS. The Bacteroidetes/Firmicutes (B/F) ratio was evaluated, and the important features for predicting sample characteristics were identified and generated as a heatmap by utilizing the random forest classifier, a machine-learning method^42^. Linear discriminant analysis (LDA) of effect size (LEfSe), multiple linear regression, phylogenetic tree analysis, and clustering heatmap analyses were performed on the web-based platform MicrobiomeAnalyst^43,44^, using the marker data profiling module. A similar rarefaction depth was maintained, resulting in the removal of one sample from the 12-week time point. For this analysis, data were filtered for low abundant features, with a minimum of 20% samples containing at least four counts. Additionally, 20% of the low-variance features, measured using the interquartile range, were removed. The data were normalized using total sum scaling (TSS). The LEfSe algorithm^45^ was employed to detect biomarker features/taxa with significant differential abundance and their effect size among different variables by employing the Kruskal–Wallis rank sum test. A multiple linear regression model with covariate adjustment was utilized to determine the association between microbial taxa and the exercise intervention group. Two-way ANOVA and Tukey’s post-hoc tests were used for calculation of statistical significance, which was set to adjusted *p*-value < 0.05, and error bars represented mean ± SEM.

## Results

The 16S metagenomics analysis of mouse fecal samples revealed a dataset of filtered quality reads generated after sequencing the V4 region of 40 DNA samples from the pre-intervention (week 0) and final (week 12) time points. The body weights of mice were monitored to support the intervention based on diet and activity. The analysis of body weight changes showed that the HFD group gained significant weight at week 7 compared to the CHD group (*p* = 0.008), and the weight of HFD mice increased consistently (*p* < 0.008) till the final time point at week 12 (*p* = 0.006), compared to the CHD group (Fig. S1). Moreover, the EXE group gained significantly less weight than the HFD (*p* = 0.01) and HFX (*p* = 0.04) groups at the final time point (Fig. S1).

### Bacterial richness and diversity profiling

**Figure 1:**
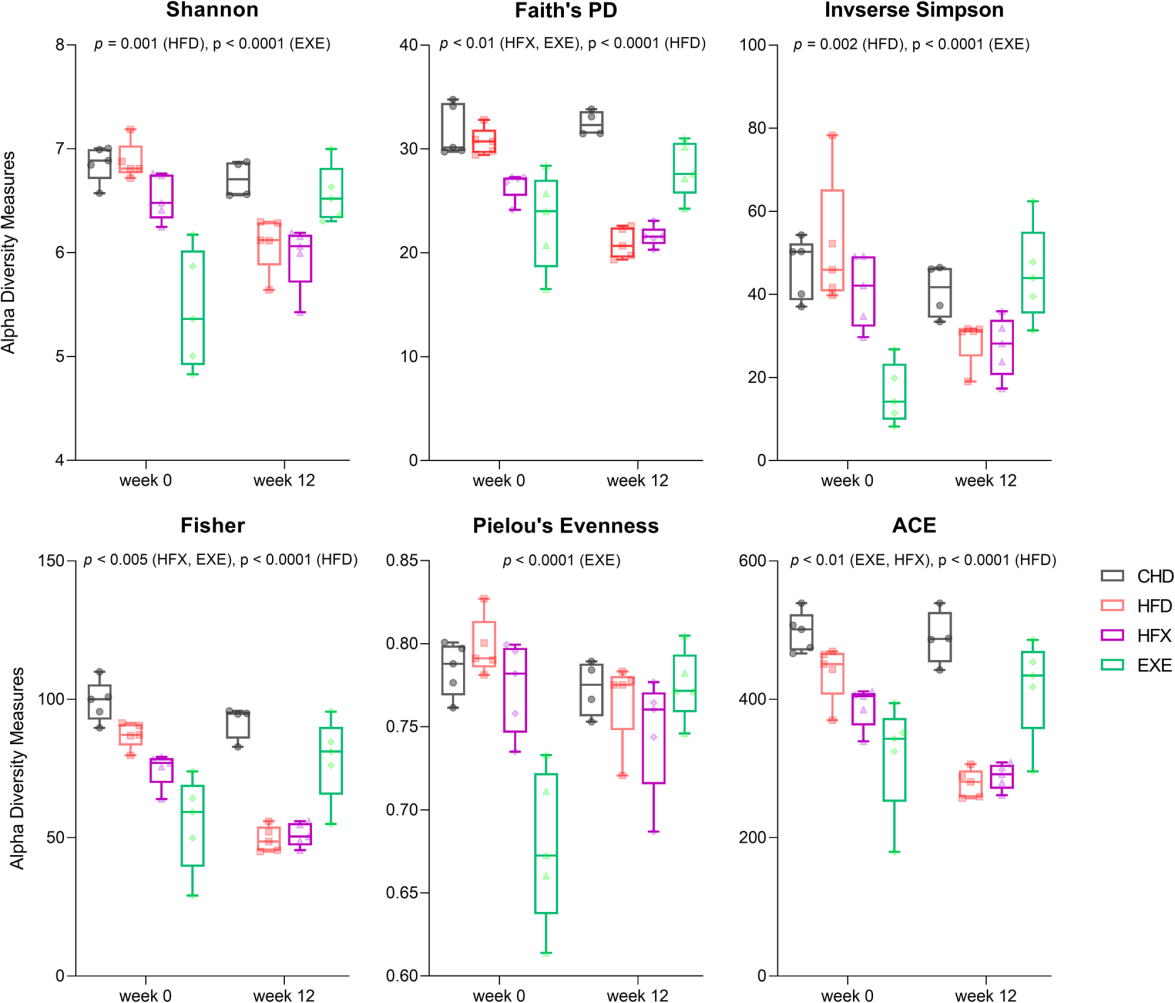
Alpha-diversity boxplots illustrating species richness (ACE) and diversity (Shannon, Faith’s phylogenetic diversity [PD], Fisher, Pielou’s Evenness and Inverse Simpson). Two-way ANOVA followed by the 2-stage linear step-up procedure of Benjamini, Krieger, and Yekutieli test and Šidák’s test were performed for mean comparison and multiple comparisons across groups. Boxes indicate interquartile ranges, lines denote medians, and whiskers demarcate the ranges. P-values are false discovery rate (FDR)-corrected. Longitudinal significance among intervention groups is marked.

Compared to the other groups, the EXE group had the lowest richness and diversity (*p* < 0.001), while the CHD, HFD, and HFX groups showed no difference pre-intervention, as estimated by Pielou’s evenness and Shannon index. ACE metric estimation during pre-intervention revealed the lowest richness in EXE group, while the other groups displayed a decreasing trend in microbial richness in the order of CHD > HFD > HFX (*p*-value range = 0.048 to < 0.0001) (Fig. 1). The alpha diversity trend showed significantly lower diversity in the EXE group than in the CHD (*p* =  < 0.001), HFD (*p* =  < 0.0001), and HFX (*p* < 0.02) groups, as measured by the Inverse Simpson and Fisher metrics, during pre-intervention. Moreover, the Fisher metric showed decreased richness in HFX compared to CHD (*p* = 0.0016). Phylogenetic diversity (Faith’s PD), too, was the lowest in the EXE group (*p* = 0.01), and it was significantly lower than the HFX group (*p* = 0.0054), as well. In addition, the CHD and HFD groups showed similar diversities in phylogenetic terms, which were significantly higher than those in the HFX and EXE (*p* < 0.005) samples when examined pre-intervention (Fig. 1).

At week 12, the diversity and richness of the chow-fed groups (CHD and EXE) were significantly higher than those of the high-fat diet-fed groups (HFD and HFX) (*p* < 0.005) based on all alpha diversity metrics except the Inverse Simpson index (Fig. 1). The EXE group had higher diversity than HFD (*p* = 0.049) and HFX (*p* = 0.027), as evaluated by the Inverse Simpson index, after 12 weeks of intervention. No differences were noted after 12 weeks of intervention in the evenness of the groups, measured using Pielou’s index. In this analysis, the alpha diversity in the gut microbiota of mice was not greatly influenced by 12 weeks of exercise among the intervention groups, especially in the high-fat diet-fed groups (HFD and HFX), as displayed in Fig. 1.

The longitudinal analysis comparing the data from week 12 and week 0 showed that the alpha diversity remained unchanged in the CHD group; however, it increased significantly in the EXE group over the period of 12 weeks (Shannon, *p* < 0.001; Faith’s PD, p = 0.008; ACE, *p* = 0.009; Inverse Simpson, *p* = 0.0001; and Fisher, *p* = 0.002). Interestingly, compared to week 0, 12 weeks of exercise also significantly increased the group’s evenness, estimated by Pielou’s metric (EXE, *p* < 0.001), whereas no changes in evenness were observed among the other groups (Fig. 1). Moreover, diversity (Shannon, Faith’s PD, and Fisher indices) and richness (ACE) were significantly reduced in the HFD (*p* < 0.005) and HFX (*p* < 0.03) groups over time (12 weeks). However, the Inverse Simpson metric showed nonsignificant diversity alterations in the HFX group over time (Fig. 1).

### Gut bacterial community structure

**Figure 2:**
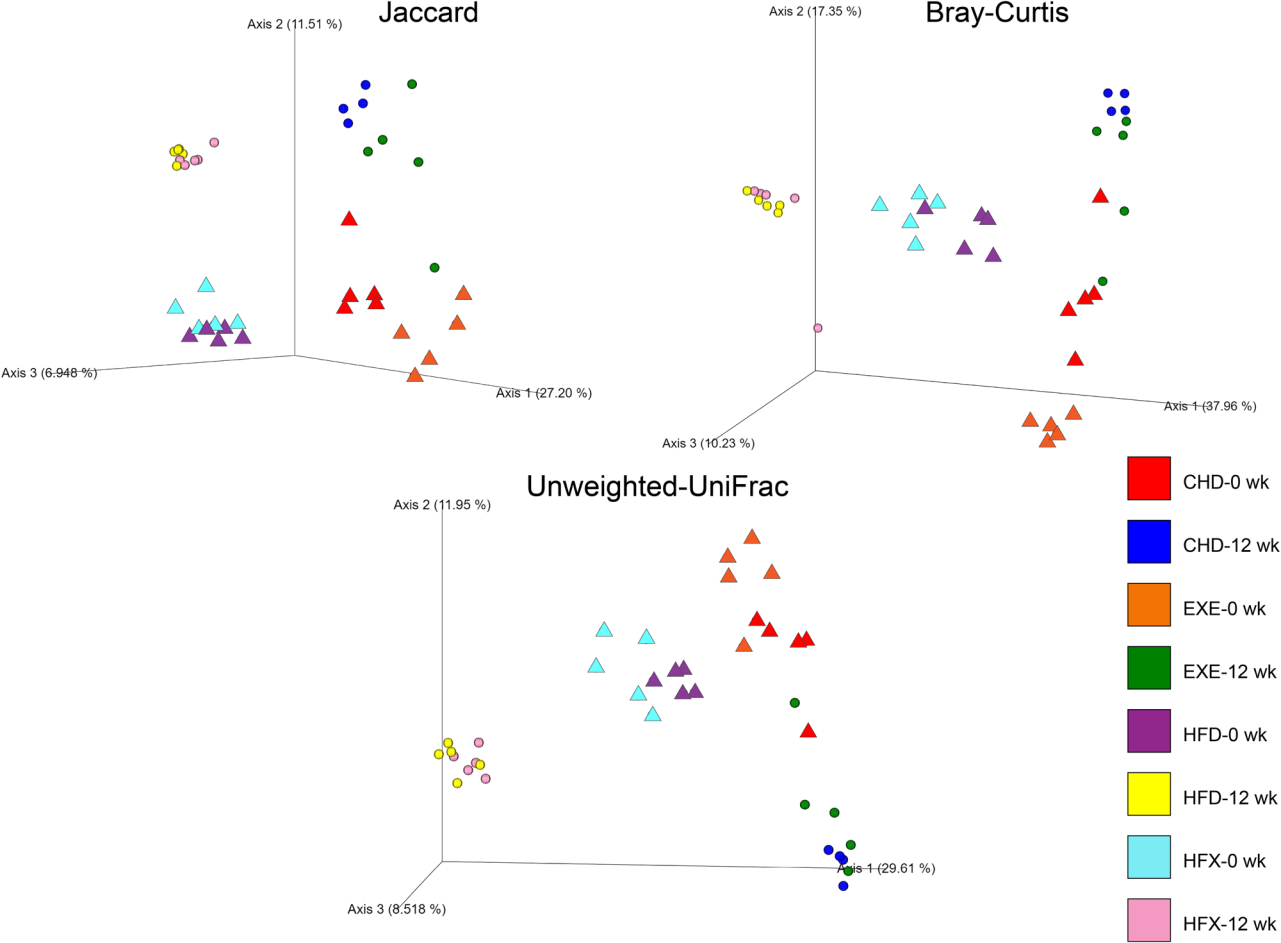
PCoA of the intervention groups using the Jaccard, Bray–Curtis, and unweighted UniFrac distance metrics based on feature level. The data points represent individual samples (triangles for 0-week and circles for 12-week). Values in parentheses show the percentage of total variance explained by each axis. Statistical significance was determined using PERMANOVA and confirmed using PERMDISP; Jaccard (*p* = 0.001), Bray–Curtis (*p* = 0.002), and unweighted UniFrac (*p* = 0.001).

Beta-diversity analysis was performed to determine the distances between microbial samples and compare the effect of the intervention on the gut microbial communities. The Jaccard index (a qualitative, nonphylogenetic metric) showed that the experimental groups did not share the exact same microbial taxa and were clustered according to the intervention categories (*p* < 0.02) at week 0 and 12. The bacterial taxa were significantly different (*p* < 0.02) among groups, and the increase in the distance among the different intervention groups at the 12-week time point, along axes 1 and 2, showed significant variation in the microbial communities (Fig. 2).

Similarly, examination of the intervention groups, based on the number of microbes and their abundance, showed that the groups did not share the same number of bacteria at the same abundances, as depicted by the Bray–Curtis dissimilarity metric shown in Fig. 2. The CHD, HFD, HFX, and EXE groups were separately clustered (*p* < 0.02) over the two time points along the axes, where axis 1 explained the maximum variance (~ 40%) in bacterial communities among the intervention groups (Fig. 2). The dissimilarity between the HFD and HFX groups was insignificant after 12-weeks of intervention, demonstrating a stronger effect of diet, compared to exercise, in the observed gut microbial community structure. Moreover, the phylogenetic relationship between bacterial taxa in the intervention groups was assessed using PCoA based on the unweighted UniFrac distance metric. Significant differences were revealed among groups (*p* < 0.02) over the two time points (Fig. 2). Overall, the variability in the beta diversity was higher in the groups initially than after 12 weeks of intervention, and the dissimilarities between the groups were evident.

The longitudinal analysis of the intervention groups generated volatility plots, displaying a divergent variation pattern along the principal coordinate axis 1, which was responsible for the maximum variability among the groups. The plots depicted the change in mean magnitude in each group based on different distance metrics, where the intervention groups deviated from each other longitudinally primarily showing an influence of diet (Fig. S2).

Adonis statistical analysis was performed to test the influence of multiple test variables on beta diversities in the gut microbiota of the mice. Diet was the strongest factor influencing the unweighted UniFrac distances, by explaining 21.3% (*p* = 0.001) of the variance, as shown in Table 1. After adjusting for diet in the Adonis model, mouse age (5 and 17 weeks) and exercise retained a statistically significant effect by explaining 11.8% (*p* = 0.001) and 4.3% (*p* = 0.017) of the overall diversity, respectively (Table 1). Similar results were obtained using the Adonis model for the Jaccard distance metric, where diet (R^2^ = 20%, *p* = 0.001), age (R^2^ = 11.4%, *p* = 0.001), and exercise (R^2^ = 3.6%, *p* = 0.046) were significantly associated with the variance (Table S1). Additionally, diet and age retained a significant influence by explaining 33.5% and 12.5% (*p* = 0.001) of the beta diversity in the mouse gut, respectively, whereas exercise showed no effect (*p* = 0.057, ns), based on the Bray–Curtis distance metric (Table S2).

**Table 1:** Adonis multivariate analysis of the unweighted UniFrac distance metric.

Factors	Df	Sums of squares	Mean squares	F. Model	R^2^	*p*-value
Exercise	1	0.209	0.209	2.429	0.043	0.017
Age	1	0.566	0.566	6.583	0.118	0.001
Diet	1	1.024	1.024	11.911	0.213	0.001
Residuals	35	3.010	0.086	–	0.626	–
Total	38	4.890	–	–	1.000	–

### Gut bacterial compositional analysis

**Figure 3:**
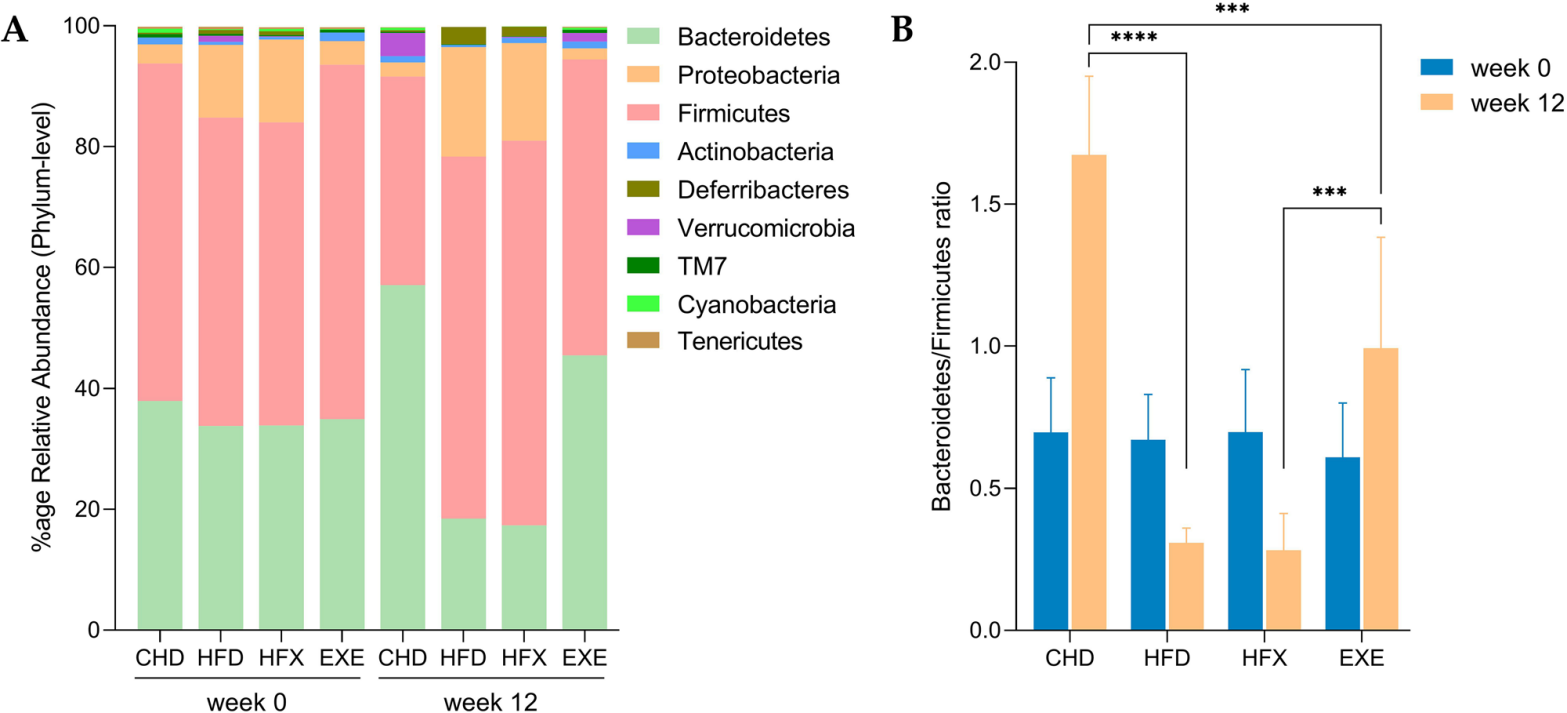
Taxonomic profiling of the gut microbiota of mice among experimental groups over time. (**A**) Phylum level composition, and (**B**) Bacteroidetes/Firmicutes ratio. Two-way ANOVA and Tukey’s multiple comparison tests (*p*: **** < 0.0001 and *** < 0.0005).

Among the nine phyla identified in the mouse gut microbiota, the two dominant phyla were Bacteroidetes and Firmicutes, which represent gram-negative and gram-positive bacterial populations, respectively (Fig. 3A). The relative abundance of Bacteroidetes increased significantly from the initial 38%, to 57%, after 12 weeks of intervention in CHD (*p* < 0.0001). Likewise, in the EXE group, the proportion of Bacteroidetes after 12 weeks increased (~ 45%) compared to week 0 (35%) (*p* < 0.0001), as shown in Fig. 3A.

The relative percentage of the members of the Firmicutes phylum significantly increased in the high-fat diet fed group (HFD: ~ 60% and HFX: 63.6%, *p* < 0.0001) after 12 weeks compared to week 0 of the intervention (~ 50%), and the proportions of this phylum in the high-fat diet fed groups also increased compared to those in the CHD and EXE groups at week 12 (34.5% and 49%, respectively, *p* < 0.0001). In contrast, the relative abundance of Firmicutes significantly decreased in CHD (*p* < 0.0001) and EXE (*p* = 0.0001) over time. The relative proportion of the Proteobacteria phylum was significantly higher in HFD (*p* = 0.0005) and HFX (*p* < 0.0001) compared to CHD initially. A similar trend was observed for HFD (*p* = 0.002) and HFX (*p* < 0.0001) compared to EXE at week 0. The expansion of Proteobacteria in the HFD and HFX groups was amplified at week 12 compared to that in the CHD and EXE groups (*p* < 0.0001) (Fig. 3A). However, the difference in the blooming of Proteobacteria in the high-fat diet-fed groups was not significant between weeks 0 and 12. In addition, no variability was observed, either among groups or temporally, in the taxonomic composition of other reported phyla, including Actinobacteria, Deferribacteres, Verrucomicrobia, TM7, Cyanobacteria, and Tenericutes.

The B/F ratio was similar among the groups at week 0 and no difference was observed between the HFD and HFX groups after 12 weeks of intervention (Fig. 3B). However, this ratio was significantly different between CHD and EXE at the 12-week time point (*p* < 0.0002). Additionally, it significantly decreased in the high-fat diet-fed groups (HFD and HFX) compared to CHD (*p* < 0.001) and EXE (*p* < 0.0003), at the 12-week time point (Fig. 3B). Over the course of 12 weeks, the B/F ratio significantly increased in the CHD (*p* < 0.0001) and EXE (*p* = 0.041) groups, whereas it decreased in the HFX (*p* = 0.023) and HFD (*p* = 0.057, ns) groups compared to that at the initial time point (Fig. 3B).

Phylogenetic tree analysis was used to assess the evolutionary relationships among taxonomic groups at the family level. Members of *Odoribacteraceae*, a family of the phylum Bacteroidetes, were present in reduced proportions in the EXE group at week 12 compared to initial time point (Fig. S3). The relative abundance of the closely placed *Porphyromonadaceae* family bloomed in the EXE samples at week 12 compared to week 0. Moreover, in the HFD group, the proportions of *Verrucomicrobiaceae* and *Prevotellaceae* shrank at the final time point compared to the initial time point (Fig. S3).

### Gut bacterial temporal variations at the genus/species level

Relative variations at the genus/species level were studied and elaborated by comparing group differences at week 0 (initial period), week 12 (final period), and over time (between weeks 0 and 12). Approximately 40–60% of the bacteria remained unclassified at the genus level.

**Figure 4:**
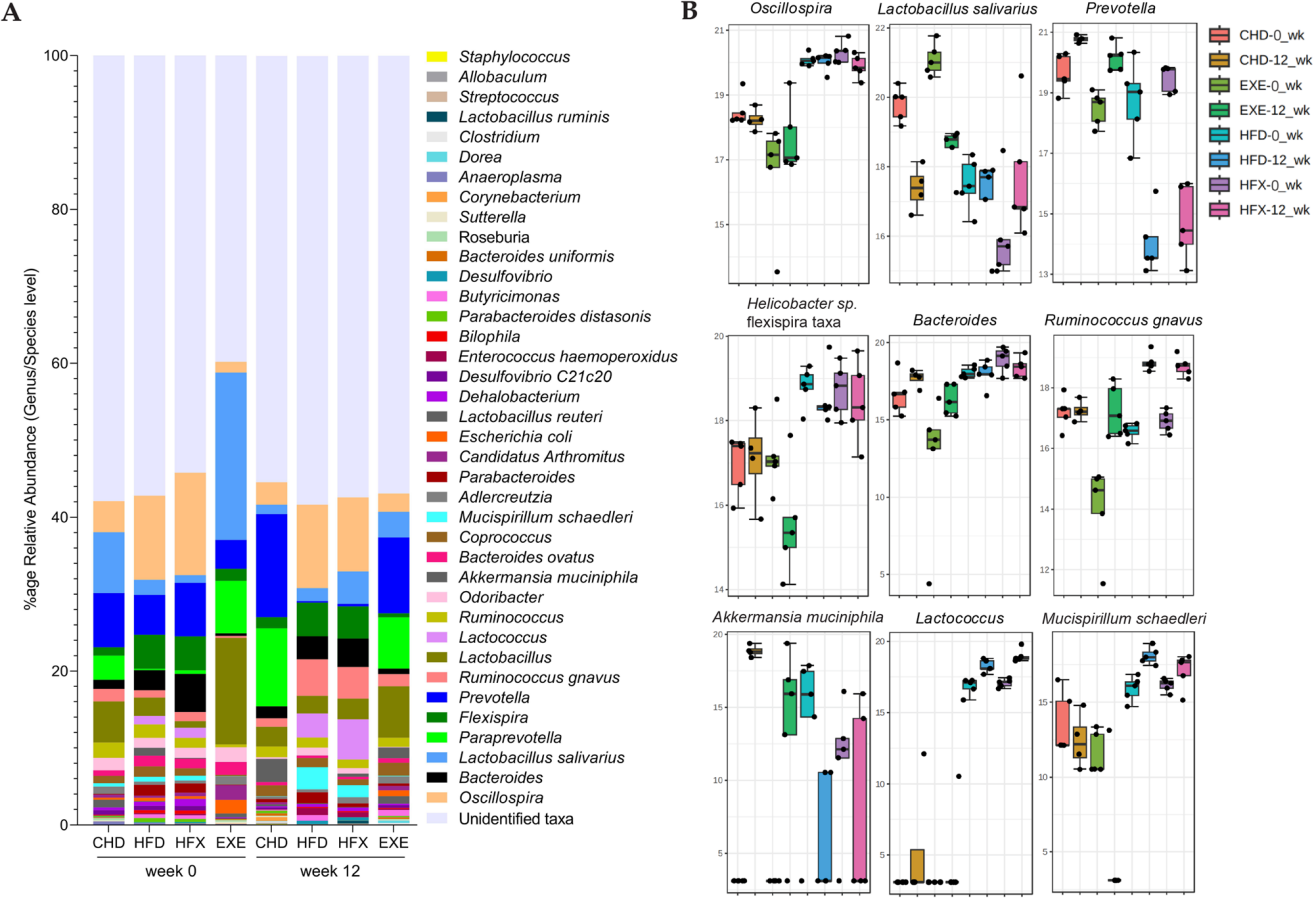
Relative abundance of microbiota at the genus/species level in the guts of mice among the intervention groups over time, comparing the initial (week 0) and final time points (week 12). (**A**) Percent relative abundance. Two-way ANOVA and Tukey’s multiple comparison tests were used to determine significance (*p* < 0.05). (**B**) Log-transformed counts of differentially abundant features estimated using the LEfSe algorithm.

The *Oscillospira* genus (Firmicutes, *Ruminococcaceae*) was significantly higher (*p* < 0.0001) in the groups fed high-fat diet (HFD and HFX) than in the chow-fed groups (CHD and EXE) at both the initial (week 0) and final time points (week 12). Moreover, the relative proportion of *Oscillospira* was initially higher in CHD (*p* = 0.006) than in EXE, however, the difference in the relative proportion of *Oscillospira* between the CHD and EXE groups became insignificant after 12 weeks of intervention (Fig. 4A,B). Longitudinal analysis showed no difference in the relative percentage of *Oscillospira* between the groups over the period of 12 weeks, except for HFX, which showed a relatively reduced proportion (*p* =  < 0.0001) after 12 weeks. The relative abundance of *Lactobacillus salivarius* (Firmicutes, *Lactobacillaceae*) in the CHD and EXE groups (*p* < 0.0001) was higher initially than in the final time point of analysis. Moreover, the EXE group had higher relative proportions of the gram-positive bacterium than CHD at the 0-week time point, but the difference between the two groups became negligible after 12 weeks. No difference was found between the HFD and HFX groups at the initial time point and between the CHD and EXE groups at the final time point. However, the *L. salivarius* abundance significantly increased in the HFX group over time (*p* = 0.0002).

Another *Lactobacillus* sp. showed a similar pattern of relative abundance; it was initially higher in the CHD (*p* < 0.002) and EXE (*p* < 0.0001) groups than in the HFD and HFX groups, whereas, at the final time point, abundance in the CHD group became similar to the HFD and HFX groups. However, the EXE group retained a significantly higher relative abundance than the groups fed the high-fat diet (*p* < 0.0001), at the final time point. The longitudinal analysis for *Lactobacillus* sp. revealed a similar reduction pattern to that of *L. salivarius* in the CHD (*p* = 0.003) and EXE (*p* < 0.0001) groups over time. In contrast, *Lactobacillus* sp. remained significantly abundant in EXE compared to CHD (*p* < 0.0001) at the final time point (Fig. 4A,B).

The relative abundance of the *Prevotella* genus (Bacteroidetes, *Prevotellaceae*) was almost doubled in the CHD (13.4%) and EXE (~ 10%) groups (*p* < 0.0001) at the final time point (week 12) compared to its abundance at the initial time point, where it was significantly lower in the EXE group compared to HFX (*p* = 0.0003) and CHD (*p* = 0.0002) (Fig. 4A,B). The levels of *Prevotella* dropped significantly in HFD and HFX (*p* < 0.0001) from ~ 5% and ~ 7%, respectively, to less than 1% over the time duration of 12 weeks. The *Paraprevotella* genus (Bacteroidetes, *Prevotellaceae*) shares the same family with *Prevotella* and showed a similar pattern of abundance in the CHD and EXE groups and negligible relative counts in the high-fat diet-fed groups (HFD and HFX) at both the initial (*p* < 0.006) and final time points (*p* < 0.0001). Moreover, *Paraprevotella* increased more than threefold in CHD (*p* < 0.0001) over time, whereas no difference was observed in EXE over time (Fig. 4A). *Flexispira* has been referred to as *Helicobacter* sp. along with the appropriate flexispira taxon number^46,47^. Therefore, we used *Helicobacter* sp. flexispira taxa to refer to the *Flexispira* genus (Proteobacteria, *Helicobacteraceae*). *Helicobacter* sp. flexispira taxa showed a higher abundance pattern in the HFD and HFX samples than in the CHD and EXE samples, at both the initial and final time points (*p* < 0.005), with no significant change over time (Fig. 4A,B).

*Bacteroides* (Bacteroidetes, *Bacteroidaceae*) relative abundance was initially higher in HFX than in the chow-diet fed groups (CHD and EXE, *p* < 0.0001) and HFD (*p* = 0.02). However, at the final time point, HFD, HFX, and CHD showed similar *Bacteroides* relative abundances, but its relative proportion in EXE was significantly reduced compared to that in the HFD (*p* = 0.03) and HFX (*p* = 0.0009) groups. No temporal variations were found among the groups for *Bacteroides* (Fig. 4). *Ruminococcus gnavus* (Firmicutes, *Lachnospiraceae*) initially showed similar abundance among the groups. However, its relative abundance significantly increased in the high-fat diet-fed groups (HFD and HFX) compared to that in the CHD (*p* = 0.002) and EXE (*p* < 0.02) groups at the final time point. Longitudinal analysis revealed a significant increase in the HFD (*p* < 0.001) and HFX (*p* = 0.001) groups—from ~ 1% to ~ 5%—over 12 weeks. Similarly, *Lactococcus* sp. (Firmicutes, *Streptococcaceae*) was significantly enhanced by ~ fivefold only in HFX (*p* = 0.0001) as assessed by the longitudinal analysis over time. Additionally, *Lactococcus* sp. counts were negligible in the CHD and EXE samples. *Akkermansia muciniphila* (Verrucomicrobia, *Akkermansiaceae*), a gram-negative symbiotic bacterium, was absent in CHD and EXE at week 0; however, it was enhanced in EXE and significantly enhanced in CHD (*p* = 0.0008), after 12 weeks of intervention. Moreover, the CHD group was significantly more abundant in *A. muciniphila* (*p* < 0.01) than the HFD and HFX groups at the final time point of analysis. The groups fed high-fat diet had negligible relative proportions of *A. muciniphila*. Among members of the gut microbiota, the relative abundance of *Mucispirillum schaedleri* (Deferribacteres, *Deferribacteraceae*) was initially (week 0) the lowest in all groups, at < 1% relative proportions; however, its abundance was significantly enhanced in HFD (*p* = 0.03) longitudinally, and, at the final time point (week 12), the species was relatively abundant in HFD compared to CHD and EXE (*p* = 0.002). No significant longitudinal or among-group statistical differences were observed in the other bacterial genera/species, as shown in Fig. 4A.

### Characteristics, biomarker features, and clustering analysis

**Figure 5:**
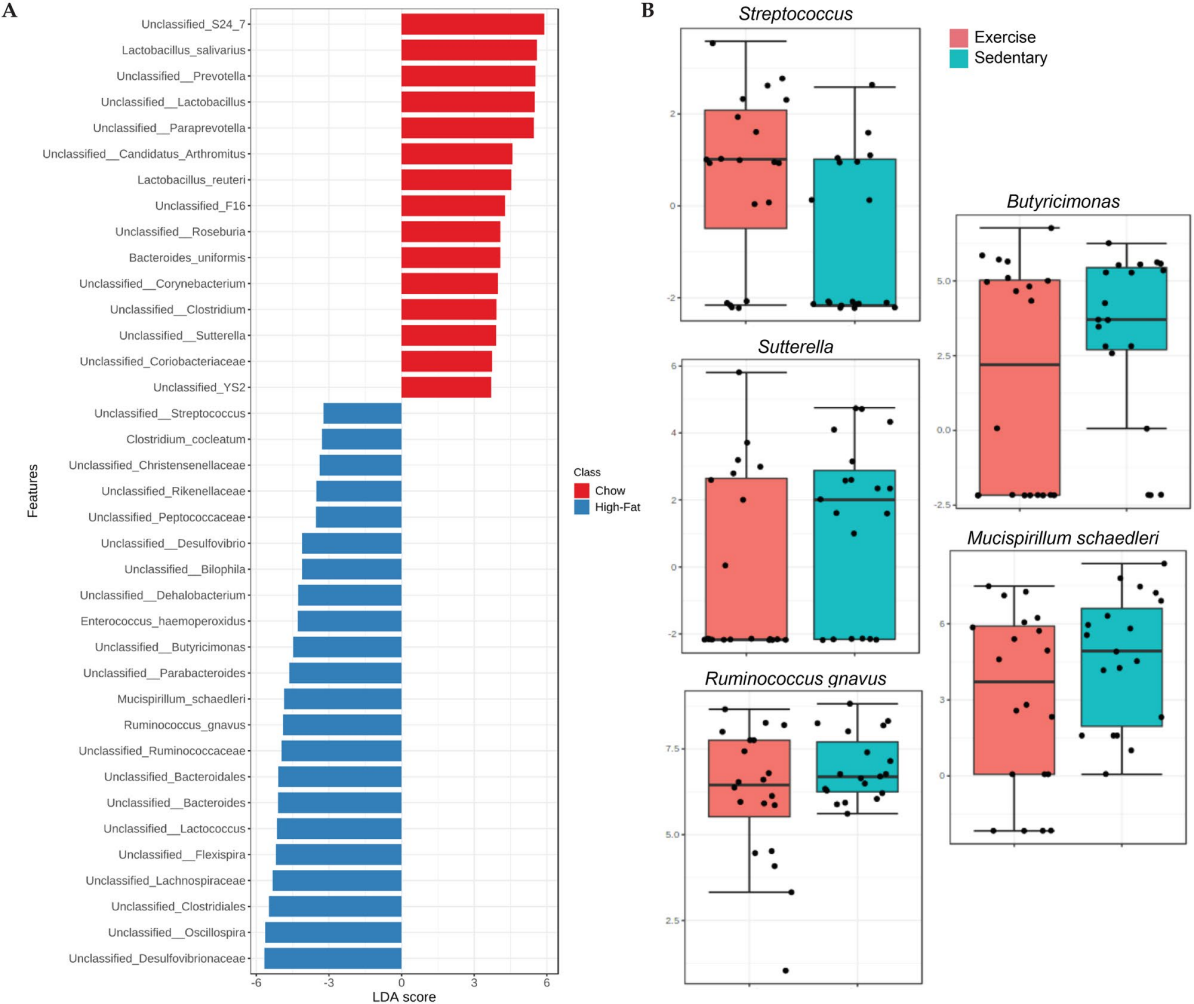
Analysis of differentially abundant bacterial taxa based on diet and exercise interventions. (**A**) LEfSe analysis based on diet is shown as a histogram of the log LDA score > 3 and FDR-corrected p value < 0.05 for the significant taxa. (**B**) Multiple linear regression analysis associating microbial genera/species with exercise intervention after covariate adjustment for diet and intervention duration. The Y-axis shows log-transformed counts of significantly altered bacterial taxa (FDR-corrected *p* < 0.05).

Biomarker taxa were identified using LEfSe. The groups fed high-fat diet were characterized by relative abundances of *Lactococcus*, *Helicobacter* sp. flexispira taxa, *Oscillospira*, *Bacteroides*, *R. gnavus*, *M. schaedleri*, *Parabacteroides* (Bacteroidetes, *Porphyromonadaceae*), *Butyricimonas* (Bacteroidetes*, Odoribacteraceae*), *Enterococcus haemoperoxidus* (Firmicutes, *Enterococcaceae*), *Bilophila* (Proteobacteria, *Desulfovibrionaceae*), *Dehalobacterium* (Firmicutes, *Peptococcaceae*), *Desulfovibrio* (Proteobacteria, *Desulfovibrionaceae*), *Streptococcus*, and *Clostridium cocleatum* (Firmicutes, *Clostridiaceae*) (Fig. 5A). The chow diet-fed groups were typified by relative abundances of *Prevotella*, *Paraprevotella*, *Candidatus arthromitus* (Firmicutes, *Clostridiaceae*), *L. salivarius*, *Lactobacillus reuteri*, *Roseburia* (Firmicutes, *Lachnospiraceae*), *Bacteroides uniformis*, *Corynebacterium* (Actinobacteria, *Corynebacteriaceae*), and *Sutterella* (Proteobacteria, *Sutterellaceae*), as illustrated in Fig. 5A.

**Figure 6:**
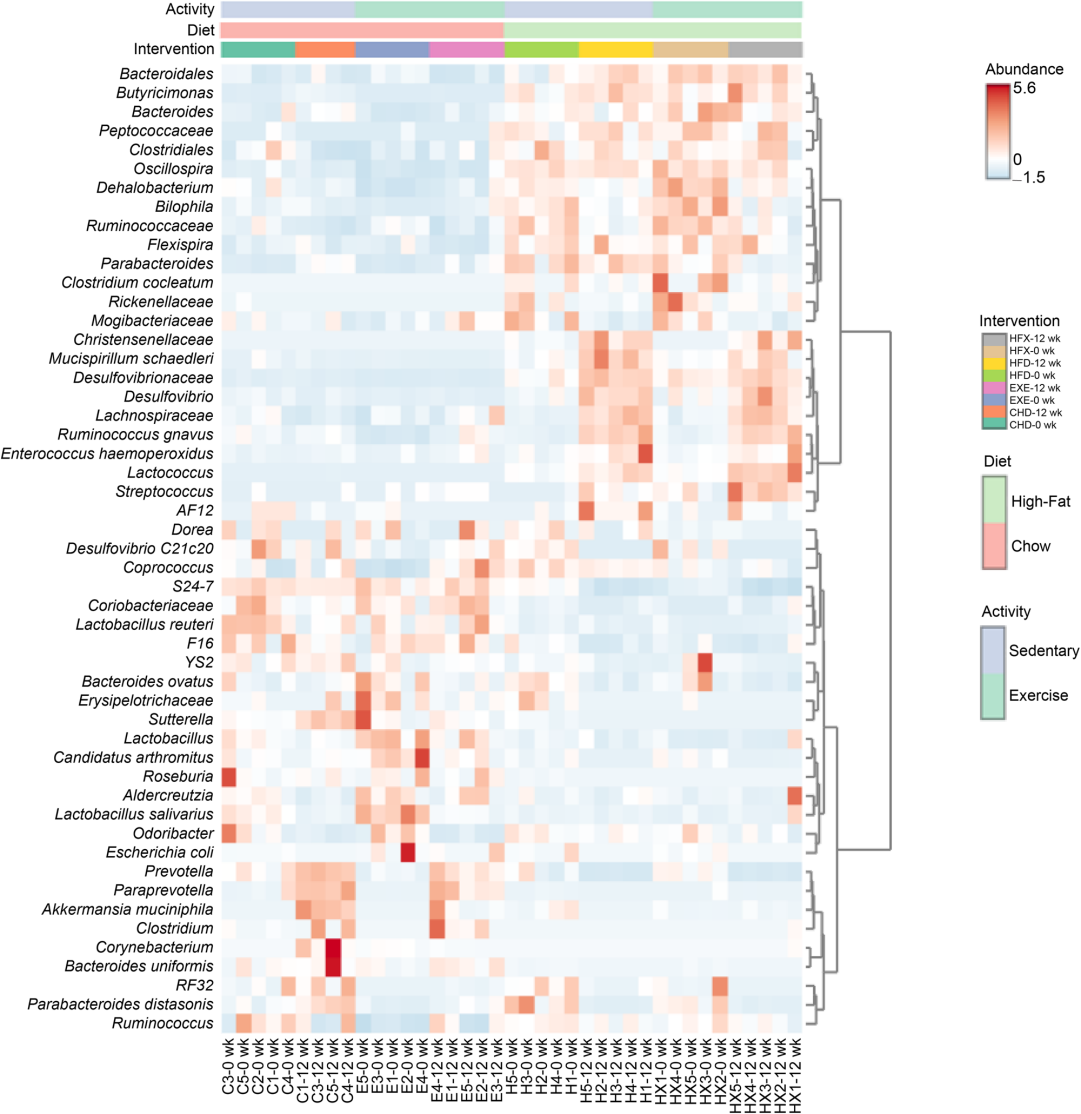
Heatmap representing hierarchical clustering analysis at the genus/species level. The analysis was performed with normalized data and autoscale feature standardization. Cluster organization was performed based on intervention group, using the Minkowski distance measure and Ward’s algorithm.

The gut bacterial taxa associated with exercise were revealed using a multiple linear regression model, after adjusting for diet and intervention duration. The relative proportions of *Sutterella* (*p* = 0.04), *Butyricimonas* (*p* = 0.01), *R. gnavus* (*p* = 0.03), and *M. schaedleri* (*p* = 0.04) were significantly reduced by the exercise intervention compared to those in non-exercise or sedentary mice. However, *Streptococcus* (*p* = 0.01) abundance was enhanced in the exercise group, as shown in Fig. 5B. The distribution of gut microbiota across samples was evaluated by visualizing the hierarchical clustering analysis heatmap, which showed that members of the *Christensenellaceae* family were enhanced in the HFD and HFX groups at week 12, whereas the unclassified *Coriobacteriaceae* (Actinobacteria) and F16 (TM7) bacterial families were abundant in the CHD and EXE groups (Fig. 6). *B. uniformis* was relatively enriched in CHD and EXE at week 12. Interestingly, the *Bilophila* genus was higher in the HFD and HFX samples at week 0 and diminished at week 12 (Fig. 6).

The random forest classifier machine learning algorithm was used to predict the most important bacterial taxa among the identified features that are predictive of the characteristics of the intervention groups. The bacterial taxa representing the family *Desulfovibrionaceae* (Proteobacteria) and most members of *Lachnospiraceae* (Firmicutes), including *Coprococcus* sp., were highly abundant in the high-fat diet intervention groups (HFD and HFX) over time and compared to the chow-diet intervention groups (CHD and EXE) (Fig. S4). Most of the species in the *Ruminococcaceae* family (Firmicutes) were enhanced in the high-fat diet intervention groups, particularly *Oscillospira* spp. *Streptococcus* sp. from the *Streptococcaceae* family (Firmicutes) bloomed in HFD and HFX over time and in comparison, to the chow-diet intervention groups (Fig. S4). Bacterial taxa of the *Coriobacteriaceae* family (Actinobacteria) were associated with the chow-diet intervention groups. Bacterial taxa belonging to the *Rikenellaceae* family (Bacteroidetes), including genus *AF12*, were relatively enhanced in the high-fat diet intervention groups compared to the chow-diet intervention groups. Moreover, two *L. reuteri* spp. from the *Lactobacillaceae* family (Firmicutes) were found to be contrastingly associated with the high-fat diet intervention groups over time, while the other bloomed in the chow-diet intervention group at the final time point (week 12) (Fig. S4).

## Discussion

The combination of a well-balanced diet and regular exercise supports metabolic health by lowering the risk of metabolic diseases like T2D and obesity. Microbiota accessible diet have significant influence on the ecology of the gut ecosystem^48^, by supporting the growth of beneficial bacteria, promoting the intestinal barrier function, lowering systemic inflammation and preventing some high-fat diet induced detrimental effects^49,50^. Our study demonstrated the longitudinal effect of CHD and HFD in relation to exercise and elaborated the influence on gut microbial changes. After the intervention the HFD mice significantly gained weight compared to CHD mice (Fig. S1). However, EXE mice did not show difference in weight compared to CHD mice (Fig. S1). This can be explained by the time-dependent effect of exercise. It is known that skeletal muscle function exhibits circadian rhythms like many other metabolic and inflammatory processes in the body. In addition, exercise performance is known to elevate in the evening overlapping with the maximum mitochondrial capacity of the skeletal muscle^51^. A study published recently showed in a disease model of mice that late dark phase (Zeitgeber time 22–23), but not early dark phase (Zeitgeber time 13–14), exercise training was able to reduce significant body fat mass (19%)^52^.

Gut bacterial richness and diversity were reduced in the high-fat diet fed mice (HFD and HFX) compared to the chow-fed mice (CHD and EXE) longitudinally (Fig. 1). Moreover, the alpha-diversity richness and evenness were temporally enhanced in EXE. Individuals with low bacterial richness have been characterized by enhanced adiposity, insulin resistance, and higher inflammation compared to individuals with high bacterial richness^53^. A systematic review showed that increased physical activity and cardiorespiratory fitness were positively associated with higher fecal bacterial alpha diversity and SCFAs^54^. A multi-omics analysis in overweight women subjected to endurance exercise (6 weeks) revealed a correlation between serum and fecal metabolites highlighting increase in serum levels of lyso-phosphatidylcholine moieties and fecal glycerophosphocholine, a signature associated with the abundance of beneficial *Akkermansia* and multiple microbial metagenome pathways^55^. The gut bacterial community structure among the high-fat diet and exercise groups was determined using different metrics. The Bray–Curtis dissimilarity index, which is sensitive to differences in abundance between species, showed significant clustering of the intervention groups longitudinally and at each time point, similar to the Jaccard distance metric-based diversity, revealing the significant effect of exercise. Our data showed significant differences in the beta diversity of the gut microbiota among the intervention groups, based on the unweighted UniFrac metric; however, the PCoA results based on weighted UniFrac were insignificant. This indicated that, although the presence or absence of bacterial features were significantly different among the groups (unweighted UniFrac, Fig. 2), most of the common taxa and features constituting the microbial community structure were not different between the groups (weighted UniFrac). The significance in unweighted UniFrac beta diversity highlights that the intervention groups significantly influence the phylogenetic diversity of the gut microbiome in a qualitative manner. The difference in beta diversity results is supported by a recent systematic review that showed higher beta diversity in athletes than in nonathletes^35^. The temporal trends were observed in the volatility plots in which the samples were segregated according to axis 1, which contributed maximally to the explained variation; this finding implies that diet is the major factor modulating gut microbiota changes (Fig. S2). Adonis, a form of one-way PERMANOVA based on unweighted UniFrac metrics, showed that, after adjusting for diet, which explained ~ 21% of the variability, age and exercise still retained a significant effect in explaining the variance by 11.8% and 4.3%, respectively (Table 1). The age of the mice, 5 weeks at the initial time point and 17 weeks at the final time point, explained 11.8% of the variance in the microbial beta diversity. Likewise, exercise duration of 12 weeks significantly explained 4.3% of the variability in the gut microbiota’s beta diversity.

As we have shown previously, diet is an important contributing factor to the changes in the gut microbiota, and significant differences can be observed in the beta-diversity of the gut microbiota in mice after eight weeks of intervention^36^. The interaction of diet with host and the gut microbiome can modulate the energy balance in humans, change beta-diversity, increase fermentation products, and affect the host enteroendocrine system^56^.

The B/F ratio is a broad term that defines any measurable difference in a single bacterial taxon to the disruption of an entire microbial community^57^ and is widely accepted to influence the maintenance of intestinal microbial homeostasis^58^. Bacteroidetes proportions have been known to decrease in people with obesity compared to those in lean people^59^. Similarly, compared to lean mice, the B/F ratio decreased in obese mice, where Bacteroidetes are reduced by 50% with a proportional increase in Firmicutes^60^. Our results corroborate those of earlier studies and showed a significant reduction in the B/F ratio (~ fivefold) in HFD compared to that in CHD after 12 weeks of intervention (Fig. 3B). A meta-analysis showed that consumption of anthocyanin rich diets improved rodent gut health by reducing obesity induced gut dysbiosis, impacting SCFA levels, and decreasing the F/B ratio^61^. In addition to diet, exercise has a positive impact on the gut microbial diversity^62^. A study showed that elite rugby players had higher gut microbial diversity than controls, and this positively correlated with the extreme exercise marker creatine kinase and a reduction in inflammatory markers^63^. Moreover, the B/F ratio was found to be inversely correlated with the total distance run, demonstrating that exercise can generate a microbial composition similar to that in lean mice and prevent diet-induced obesity in mice^64^. Furthermore, compared to adults, juvenile exercise can increase the B/F ratio, influence more genera, and increase lean body mass, revealing that host metabolism can be adaptively altered by stimulating the development of gut bacteria^65^. Similarly, our data showed a significant longitudinal effect of exercise (EXE, *p* < 0.0005) in enhancing the B/F ratio. Firmicutes taxa such as *Lactobacillaceae, Ruminococcaceae* and *Lachnospiraceae* can produce SCFA including butyrate^66^. Although majority of the SCFA producers are beneficial bacteria, butyrate can be produced by both commensal and the pathogenic bacteria through distinct and divergent pathways resulting in beneficial and/or harmful byproducts (eg. ammonia) depending upon the utilized substrates^67^.

Moreover, the substantial increase of Proteobacteria in the high-fat diet-fed groups compared to that in the chow-diet intervention corroborates earlier reports^36,68^. The B/F ratio was not changed between the HFD and HFX groups at the final time point, revealing no effect of exercise in the high-fat diet intervention groups (Fig. 3B).

The relative abundance of *Oscillospira* was found to be significantly enhanced in the HFD groups (Figs. 4, 5A, 6). *Oscillospira* and interleukin (IL)-10 levels have been linked to gut pathophysiology and microbiota alterations in diet-induced obesity, along with intestinal paracellular permeability as potential early dysfunctions in the gut that might lead to metabolic disorders and obesity^69^. Higher *Oscillospira* and *Ruminococcus* abundances and lower *Barnesiellaceae* and *Christensenellaceae* abundances are considered predictors of physical frailty and sarcopenia^70^. *Candidatus arthromitus* are segmented filamentous bacteria (SFB), also designated as *Candidatus Savagella.* This commensal bacterium was originally described in the rodent intestinal tract^71^, and a genetically distinct SFB variant exists in humans^72^. SFB have implications for host immunomodulation by stimulating the differentiation and enhancement of the Th-17 cell lineage that produces IL-17 and protects the host against bacterial and fungal infections, primarily at the mucosal surface^73,74^. This bacterium was differentially abundant in the chow-fed groups (Fig. 5A). The decrease in the relative abundance of *Odoribacteraceae* in the EXE group, as shown in the phylogenetic tree analysis (Fig. S3), is supported by an earlier study in an older population, where individuals with overweight who exercised frequently showed significantly reduced proportions of the bacterial family *Odoribacteraceae*^75^. In our analysis, the *Porphyromonadaceae* family, which is placed close to *Odoribacteraceae* in the phylogenetic tree, was found to be increased in the EXE group at the final time point (Fig. S3). A gut microbiota biomarker study in Italian adults associated decreased proportions of *Porphyromonadaceae* (Bacteroidetes) to people with overweight or patients with obesity compared to normal weight controls^76^, explaining the apparent beneficial increase of the bacterial family in the EXE group in the current study.

*Bilophila* was associated with the high-fat diet group in the LEfSe analysis, and clustering analysis revealed that it was abundant only at the initial time point, while its relative abundance was low at the final time point (Figs. 5A, 6). A study of the gut microbiota composition of athletes reported a greater abundance of *Bilophila* in high-performing individuals^77^; this is partially contrasting with our data where *Bilophila* appears to be abundant in the HFD group. *Bilophila wadsworthia* has been reported to synergize with HFD to enhance inflammation, intestinal barrier dysfunction, and disrupted metabolism of bile acid, leading to glucose dysmetabolism and hepatic steatosis^78^. The exercise-related study described the genus, but not the species name, likely explaining the disparity in results.

The data analyzed using taxonomic ranks and a random forest supervised machine learning algorithm, showed that *Oscillospira*, *R. gnavus*, *Helicobacter* sp. flexispira taxa, *Lactococcus*, *Butyricimonas*, *Streptococcus*, and members of *Lachnospiraceae*, *Ruminococcaceae*, *Desulfovibrionaceae*, and *Rikenellaceae* were highly abundant in the high-fat diet-fed groups (Fig. S4). Interestingly, 12 weeks of exercise significantly reduced *Oscillospira* proportions in HFX. These findings are supported by a recent study where *Oscillospira*, *Ruminococcus*, and members of *Lachnospiraceae* and *Ruminococcaceae* have been reported to be associated with high-fat diet-induced obesity, implying gut microbiota dysbiosis^79^. Another study corroborating our data showed an increased relative abundance of Firmicutes, such as *R. gnavus* and *Streptococcus* sp., in Italian adults with obesity, confirming that body fat is positively associated with Firmicutes and negatively associated with muscle weight and/or physical activity^76^.

*R. gnavus* and other bacterial spp. have been linked to gut dysbiosis, BMI, cognitive decline, and inflammatory conditions, including IBD, eczema, coronary artery, and other obesity-related diseases^80–87^. Moreover, a pilot investigation showed that *Butyricimonas* and *R. gnavus* are likely to be involved in the development of chronic metabolic disorders such as T2D^88^. *Ruminococcus* has also been negatively associated with metabolites involved in glutamate and tryptophan metabolism, as well as branched-chain amino acid (BCAA), fatty acids and purines, all of which are associated with amino acid and the lipid pathways. Furthermore, *Parabacteroides* (another biomarker taxa of HFD group) has been negatively associated with metabolites involved in the arginine-proline and dihydroxy fatty acid pathways. These taxa were found relatively high in abundance in mice with higher liver triglycerides, glucose and higher adiposity^89^, which is in concordance with our data. Additionally, *Desulfovibrionaceae* and *Rikenellaceae* have been reported to be relatively abundant in HFD^90^. The Rikenellaceae bacterial family has been strongly and positively connected with tryptophan and tyrosine metabolism, whereas it showed a negative association with BCAA metabolism. The abundance of this family has been associated with diets exhibiting worse metabolic outcomes in mice^89^.

Another bacterium, *M. schaedleri* (family *Deferribacteraceae*), belonging to the Deferribacteres phylum with low abundance, has been suggested to be a pathobiont. In the current study, it was significantly enhanced in the HFD group longitudinally (over 12 weeks) (Figs. 4, 5A, 6). This gut commensal is an inhabitant of the intestinal mucus layer and has been associated with inflammation^91^. A recent report causatively associated *M. schaedleri* to the development of Crohn’s disease-like colitis in immunocompromised mice^92^. HFD is known to induce low-grade chronic inflammation^93,94^, and the possible association of *M. schaedleri* with HFD lies in its capacity to deal with inflammation. *M. schaedleri* handles oxidative stress by utilizing specialized scavenging systems for reactive oxygen species (ROS) and oxygen and by expressing secretion systems (type IV) and effector proteins. It can alter mucosal gene expression in the host, leading to bacterial expansion during inflammation^95^. These host–bacteria interactions may contribute to the susceptibility of the host and influence a disease phenotype. Our data revealed a negative association between exercise and *R. gnavus*, *Butyricimonas*, and *M. schaedleri*, which is encouraging and supported by an earlier study that showed a significantly lower relative abundance of *R. gnavus* in exercise-trained mice^96^.

The study has the limitation of the small sample size which poses difficulty to account for the inter-mice inherent variability and determining significance. Moreover, the running velocity of the final 20 m/min is a critical speed for the C57BL/6 J mice and is also challenging for the wider human population. The study involved female mice to reduce data variability, even though females seems less vulnerable than males to the effect of HFD-induced obesity on weight gain, metabolic alterations and neuronal plasticity^97^. We were able to identify genus level variations as a result of the interventions, and studies can be designed in future to study the effects of the taxa on the metabolic signature and immune response of the host in correlation with the microbiome.

Quantifying temporal variations in the gut microbiota is pivotal for determining the association between bacterial taxa/species and an underlying metabolic condition that cannot be identified otherwise. This can lead to the application of microbiome evaluations in diagnostics by benchmarking the host microbiota against signature bacterial taxa. Moreover, mechanistic studies related to microbes of importance can yield an understanding of the molecular players and identification of the signaling pathways involved in the development of a disease or disorder. However, we believe that identifying causative relationships between gut commensals and various high-fat diet-induced metabolic conditions can suitably foster the development of targeted microbiome therapeutics to manage chronic diseases in the future.

In conclusion, the longitudinal study revealed the exercise and diet induced variations in the gut bacterial community structure, diversity, and local stability. B/F ratio, alpha diversity and richness were significantly decreased in the high-fat diet groups regardless of exercise activity. Moreover, the community structure showed significant clustering into the intervention groups, illustrating an effect of exercise, in addition to diet. The alpha diversity and evenness of the exercise group was significantly enhanced over time. Moreover, the majority of variance in unweighted UniFrac and Jaccard distances was explained by diet, followed by age (time duration), and exercise factors. Moreover, *Oscillospira* was significantly higher in high-fat diet groups. Interestingly, 12 weeks of exercise temporally reduced the relative abundance of *Oscillospira* in HFX. In addition, the biomarker taxa in groups fed high-fat diet, included *Lactococcus*, *Helicobacter* sp. flexispira taxa, *Bacteroides*, *R. gnavus*, *M. schaedleri*, *Parabacteroides*, *Butyricimonas*, *E. haemoperoxidus*, *Bilophila*, *Dehalobacterium*, *Desulfovibrio*, *Streptococcus*, and *C. cocleatum*. Among these taxa, exercise significantly decreased the relative proportions of *Butyricimonas*, *R. gnavus*, and *M. schaedleri.*

### Supplementary Information

Supplementary Information.

## Data Availability

The data generated during this study are available from NCBI, in the Sequence Read Archive repository, under the BioProject PRJNA971356.
