# Supplementary material for: Temporal variations in the gut microbial diversity in response to high-fat diet and exercise
Source: Sci Rep. 2024 Feb 8;14:3282. doi: 10.1038/s41598-024-52852-4 (PMC10853223; doi:10.1038/s41598-024-52852-4)
Supplement: Supplementary file 1 — Supplementary Information. [file 41598_2024_52852_MOESM1_ESM.doc]

**Supplementary Information**

**Temporal variations in the gut microbial diversity in response to high-fat diet and exercise**

**Saba Imdad ^1,2^, Byunghun So ^1^, Junho Jang ^1^, Jinhan Park ^1^, Sam-Jun Lee ^3^, Jin-Hee Kim ^2^,*, Chounghun Kang ^1,4^,***

^1^Molecular Metabolism in Health & Disease, Exercise Physiology Laboratory, Sport Science Research

Institute, Inha University, Incheon 22212, South Korea, sb.imdad22@gmail.com (S.I.), [sportshun@gmail.com](mailto:sportshun@gmail.com) (B.S), [jangju2489@gmail.com](mailto:jangju2489@gmail.com) (J.J), [sportsjinhan@gmail.com](mailto:sportsjinhan@gmail.com) (J.P).

^2^Department of Biomedical Laboratory Science, College of Health Science, Cheongju University, Cheongju 28503, South Korea.

^3^Department of Sport Rehabilitation, College of Health, Welfare, and Education, Tong Myong University, Busan 48520, South Korea, [anada23@tu.ac.kr](mailto:anada23@tu.ac.kr) (S.-J.L.).

^4^Department of Physical Education, College of Education, Inha University, Incheon 22212, South Korea.

***** Correspondence: ck@inha.ac.kr (C.K.), [jinheekim@cju.ac.kr](mailto:jinheekin@cju.ac.kr) (J.-H.K.)

**Contents:**

**Figures**

**Figure S1.**

Longitudinal analysis of the changes in body weight of mice.

**Figure S2.**

Volatility plots demonstrating PCoA-based longitudinal analyses along axis 1.

**Figure S3.**

Phylogenetic tree analysis of the gut microbial bacteria at family level, generated using MicrobiomeAnalyst.

**Figure S4.**

Random forest classifier predicting top 100 important taxa of the intervention groups.

**Supplementary Tables**

**Table S1.** Adonis multivariate analysis of Jaccard distance metric

**Table S2.** Adonis multivariate analysis of Bray-Curtis distance metric


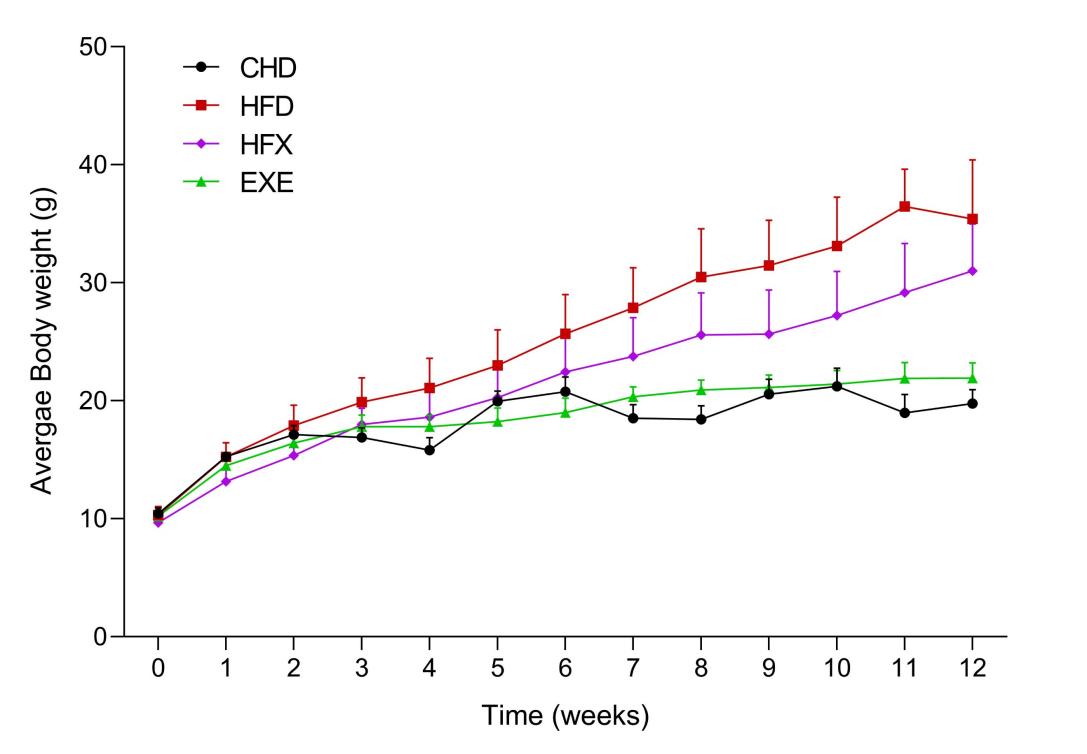


**Figure S1**. **Longitudinal analysis of the changes in body weight of mice.** Data analysis was performed using two-way ANOVA by fitting the mixed-effect model, followed by Tukey’s post-hoc test to determine significance (p < 0.05) among experimental groups over time.

**
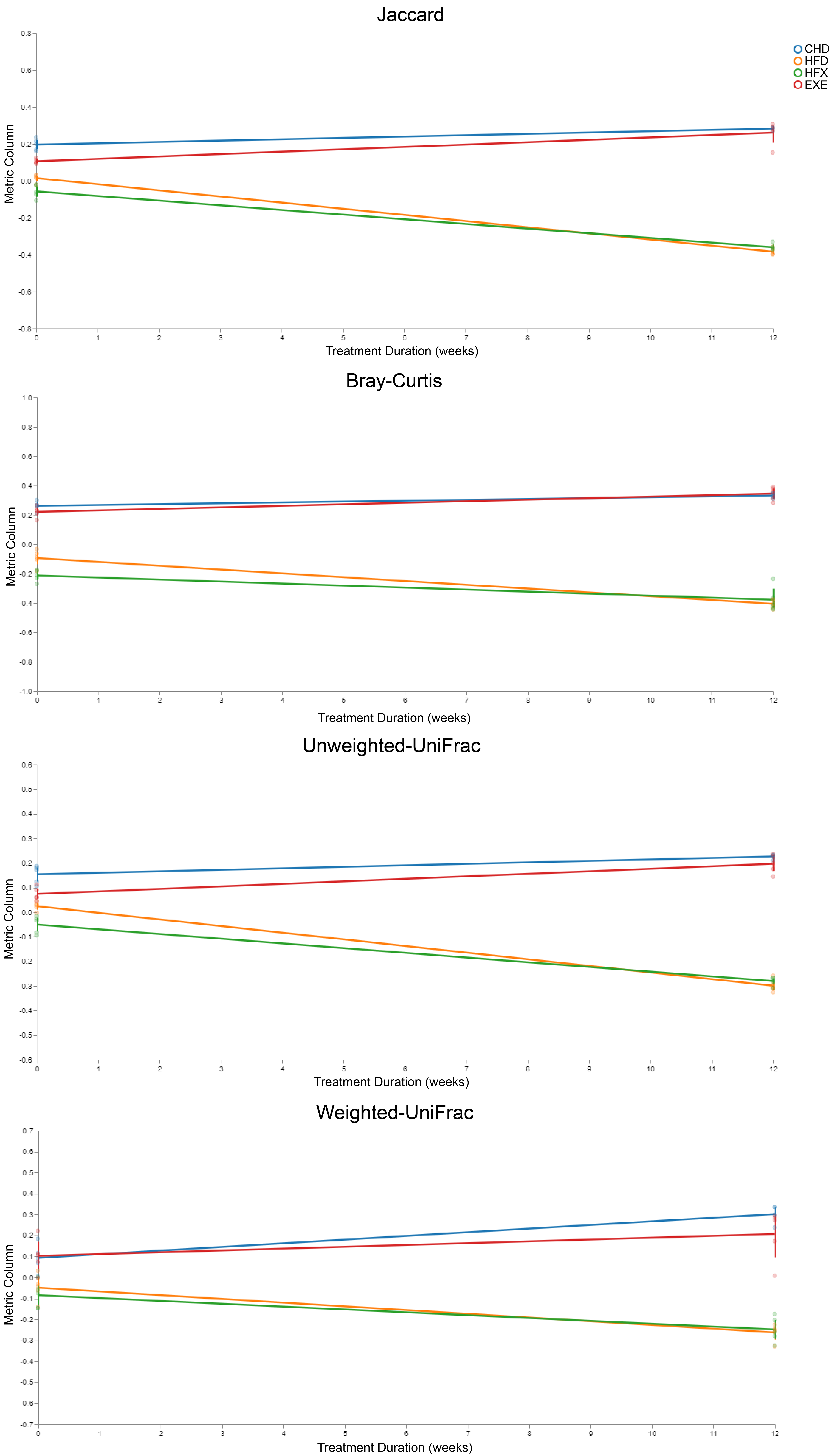
**

**Figure S2**. **Volatility plots demonstrating PCoA-based longitudinal analyses along axis 1**. The plots are based on Jaccard, Bray-Curtis, Unweighted and Weighted UniFrac distance metrics. The thick lines denote the mean changes in the distance metric over time, while the circles show the spaghetti scattering of samples in each intervention group. Error bars denote mean ± SEM.


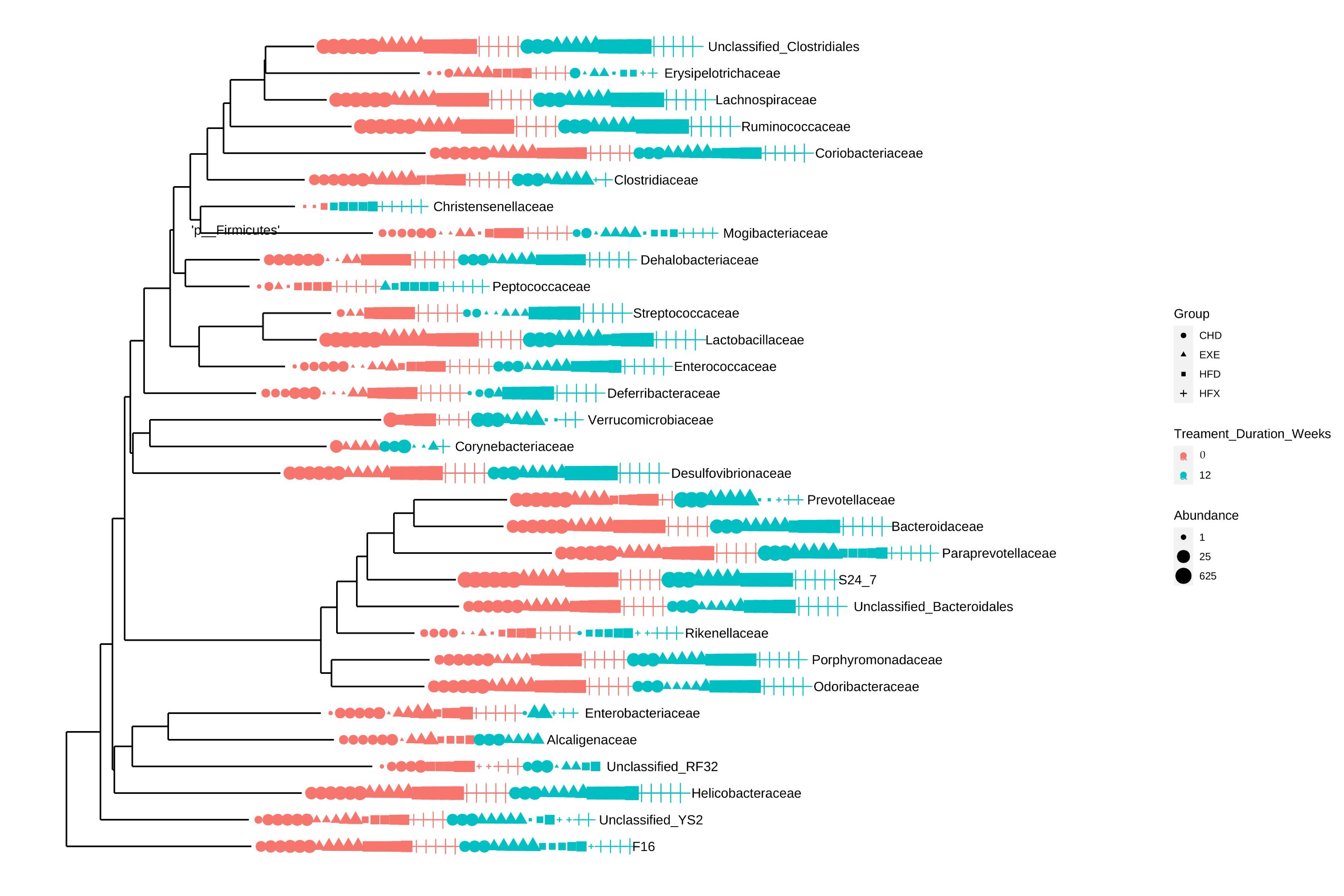


**Figure S3**. **Phylogenetic tree analysis of the gut microbial bacteria at family level, generated using MicrobiomeAnalyst.**

0

**
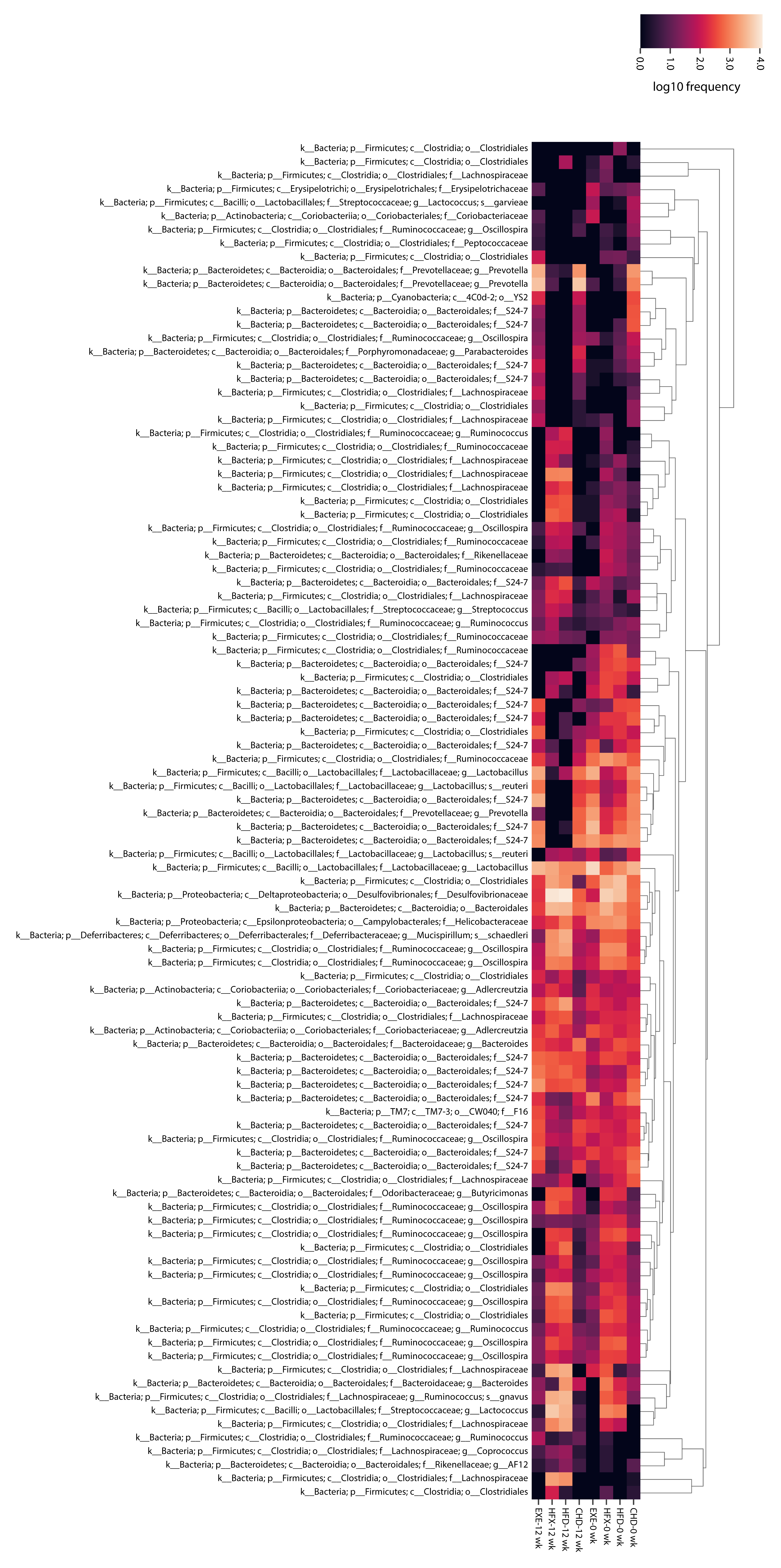
**

**Figure S4**. **Random forest classifier predicting top 100 important taxa of the intervention groups.** The overall accuracy of the model was 75 %, higher than the baseline accuracy (12.5 %).

**Supplementary Tables**

**Table S1. Adonis multivariate analysis of Jaccard distance metric.**

| **Factors** | **Df** | **Sums of Squares** | **Mean Squares** | **F.Model** | **R^2^** | ***p*-value** |
| --- | --- | --- | --- | --- | --- | --- |
| Exercise | 1 | 0.308 | 0.308 | 1.929 | 0.035 | 0.046 |
| Age | 1 | 0.975 | 0.975 | 6.105 | 0.113 | 0.001 |
| Diet | 1 | 1.717 | 1.717 | 10.748 | 0.199 | 0.001 |
| Residuals | 35 | 5.591 | 0.159 | - | 0.650 | - |
| Total | 38 | 8.592 | - | - | 1.000 | - |

**Table S2. Adonis multivariate analysis of Bray-Curtis distance metric.**

| **Factors** | **Df** | **Sums of Squares** | **Mean Squares** | **F.Model** | **R^2^** | ***p*-value** |
| --- | --- | --- | --- | --- | --- | --- |
| Exercise | 1 | 0.290 | 0.290 | 2.165 | 0.031 | 0.057 |
| Age | 1 | 1.151 | 1.151 | 8.596 | 0.124 | 0.001 |
| Diet | 1 | 3.086 | 3.086 | 23.035 | 0.334 | 0.001 |
| Residuals | 35 | 4.688 | 0.133 | - | 0.508 | - |
| Total | 38 | 9.216 | - | - | 1.000 | - |
